# Electroacupuncture Ameliorates Cognitive Deficit and Improves Hippocampal Synaptic Plasticity in Adult Rat with Neonatal Maternal Separation

**DOI:** 10.1155/2018/2468105

**Published:** 2018-03-29

**Authors:** Lili Guo, Ximin Liang, Zhanmou Liang, Xilin Liu, Jiang He, Yuanjia Zheng, Lin Yao, Yongjun Chen

**Affiliations:** ^1^South China Research Center for Acupuncture and Moxibustion, Medical College of Acu-Moxi and Rehabilitation, Guangzhou University of Chinese Medicine, Guangzhou 510006, China; ^2^School of Pharmaceutical Sciences, South China Research Center for Acupuncture and Moxibustion, Guangzhou University of Chinese Medicine, Guangzhou 510006, China

## Abstract

Exposure to adverse early-life events is thought to be the risk factors for the development of psychiatric and altered cognitive function in adulthood. The purpose of this study was to investigate whether electroacupuncture (EA) treatment in young adult rat would improve impaired cognitive function and synaptic plasticity in adult rat with neonatal maternal separation (MS). Wistar rats were randomly divided into four groups: control group, MS group, MS with EA treatment (MS + EA) group, and MS with Sham-EA treatment (MS + Sham-EA) group. We evaluated the cognitive function by using Morris water maze and fear conditioning tests. Electrophysiology experiment used in vivo long-term potentiation (LTP) at Schaffer Collateral-CA1 synapses was detected to assess extent of synaptic plasticity. Repeated EA stimulation at* Baihui *(GV 20) and* Yintang* (GV 29) during postnatal 9 to 11 weeks was identified to significantly ameliorate poor performance in behavior tests and improve the impaired LTP induction detected at Schaffer Collateral-CA1 synapse in hippocampus. Collectively, the findings suggested that early-life stress due to MS may induce adult cognitive deficit associated with hippocampus, and EA in young adult demonstrated that its therapeutic efficacy may be via ameliorating deficit of hippocampal synaptic plasticity.

## 1. Introduction

Since the early 90s in 20th century, mother-infant separation shortly after birth in hospitals has become routine to human [1] and mother-newborn repeat separation has been also widespread for a number of postpartum women who devoted themselves to work after delivery under social pressure or other social issues [2, 3]. In China, “left behind children” (LBC) living apart from their parents annually is quite a prevalent phenomenon in the last decade; the prevalence of LBC is 37.7% as reported nationally in 2013 [4]. Increasing evidences show that maternal separation (MS), as an early-life adverse event in humans, has long-term negative repercussions on child neuron development and increases vulnerability to stress-related psychopathology in adult life [5, 6].

Neonatal MS of rat is a successfully neurodevelopmental model used to mimic physiology and behavior responsiveness to early-life stress in human [7–9]. Previous studies showed that early MS may perturb neurotransmitter system in rat brain [10], increase stress reactivity in neuroendocrine signs [11], alter function of hypothalamic-pituitary-adrenal (HPA) axis [12], and express abnormal behavior phenotypes including anxiety, depression, autism, and social deficits [10, 13, 14], and impair cognitive function associated with hippocampal or prefrontal cortex [15–17], whose effects may persist and endure into adulthood.

Currently, no drugs have been proven effective in the treatment of cognitive impairment, except for encouraging patients to engage in mental or physical activity [18]. Therefore, complementary and alternative therapies for the treatment of various cognitive deficits are urgently required. Acupuncture, as a traditional therapy originating from ancient China, has become popular and been widely accepted in many countries around the world. Acupuncture therapy has been used to treat cognitive impairment of stroke and some neurodegenerative disorders including Alzheimer disease and dementia [19–23].* Baihui *(GV 20) is located above the apex auriculate, on the midline of the head, and* Yintang* (GV 29) is located at the midpoint between the two eyes [24]. In traditional Chinese medicine, GV 20 and GV 29 are the two acupoints which have been used in neurological diseases like depression, mania, epilepsy, and headache [25–27]. However, we do not know whether the cognitive impairment resulting from maternal separation in early-life could be attenuated by EA treatment in young adults. In the present study, we investigated the therapeutic efficacy of EA against cognitive impairment of young adult rats exposed neonatal MS by behavior studies including Morris water maze and fearing conditioning test.

Hippocampal synaptic plasticity is thought to be a cellular mechanism of memory formation and impaired long-term potentiation (LTP) of excitatory synaptic transmission contributes to cognitive deficits [28–30]. In the current study, electrophysiological study by using LTP recording in vivo at Schaffer Collateral-CA1 (SC-CA1) synapses in the hippocampus was to explore synaptic plasticity for the underlying mechanisms. To the best of our knowledge, our study is the first to provide evidence that EA ameliorates cognitive deficit and improves synaptic plasticity in adult rat with neonatal MS experience.

## 2. Materials and Methods

### 2.1. Experimental Animals

Male and nulliparous female Wistar rats of 2 months old were obtained from the Guangdong Medical Laboratory Animal Center. All rats were housed in groups of four with the same gender in home cages made from Plexiglas with sawdust (42 × 26 × 15 cm), in a controlled environment with temperature rooms (23 ± 2°C) and humidity maintained (50 ± 5%). The rats were under a controlled 12/12 h light/dark cycle with light on from 7:00 a.m. to 19:00 p.m. After a week of adaptation, male and nulliparous female Wistar rats were put together in standard cages with access of food and water ad libitum. After two weeks, the female rats were checked twice daily for delivery with male rats removed away.

### 2.2. Experimental Design

**Figure 1:**
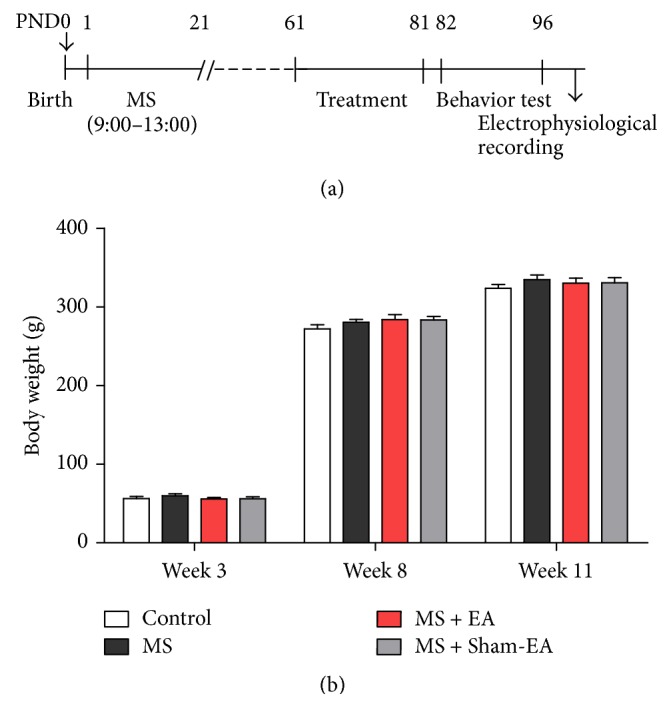
Experimental schedules and body weight gain of rats in all subgroups. (a) The experimental schedule of MS, acupuncture stimulation, behavioral tests, and electrophysiology recording. (b) Animals in all subgroups were weighted on the weaning day in the postnatal 3rd week, the day before EA treatment in the postnatal 8th week, and the day after treatment in the postnatal 11th week. There was no difference on weight among groups observed, *F*_(3,40)_ = 0.711; *P* > 0.05; *n* = 11/group; two-way ANOVA.

Wistar dams and their litters were assigned either to control group (Control) or to the mother separation group. For each litter, the day of birth was named as postnatal day 0 (PND 0). Maternal separation procedure: maternal separation took place according to the pervious protocol [31–33] (Figure 1(a)). In brief, the mothers were daily removed into another cage for four hours (9:00–13:00) and the litters remained in the home cage. After 4 hours, the mothers were returned back to their home cage. During the separation, litters were maintained in heating plate and water was provided to maintain temperature and humidity. The separation procedure last from PND1 to PND20. The mothers and litters in control were not disturbed until weaning, except for cage cleaning at PND10. All cages were cleaned firstly at PND10. Pups were weaned at PND 21 when female pups were removed, and male pups were left for experiment and housed by four or five per cage until adult age. Food and water were available ad libitum. The experimental procedure was approved by the Animals Care and Use Committee of Guangzhou University of Traditional Chinese Medicine. All efforts were made to minimize animals suffering and reduce the number of animals used for experiments.

At the PND60, the maternal separation group rats were assigned randomly into three groups: maternal separation group (MS), electroacupuncture treatment group (MS + EA), and sham electroacupuncture treatment group (MS + Sham-EA). There were forty-four rats in total, *n* = 11 rats/group.

### 2.3. Treatment

All the treatments were performed from PND61 to PND81.

#### 2.3.1. EA Stimulation

Rats in the MS + EA were anesthetized with isoflurane (RWD, Shenzhen, China). Anesthetized concentration was maintained at 2% and positioned on a stereotaxic frame (RWD, Shenzhen, China). Disposable acupuncture needles (0.25 × 13 mm, Suzhou Medical Appliance Factory, Suzhou, China) were inserted to a depth of 5 mm at the GV 20 and GV 29 after skin had been cleaned with alcohol swabs. And then a Master-8 Stimulator (Master-8, AMPI, Israel) was connected, and the electrical current was delivered to the needles. The output parameters were set as follows: frequency was held constant at 2 Hz and intensities of 2 mA, for 20 min. EA stimulation was administered every other day for 20 min starting at 8:30 a.m.

#### 2.3.2. Sham-EA Procedure

Rats in MS + Sham-EA group were anesthetized with isoflurane as MS + EA group. After the skin was cleaned with alcohol swabs, rats in MS + Sham-EA group received no electrical stimulation: disposable acupuncture needle was pasted at the surface of GV 20 and GV 29 and the needle touched the skin but not inserted the acupoints. Control group rats were anesthetized with isoflurane as MS + EA group.

### 2.4. Body Weight Measurements

To assess the effect of EA and MS on rat, body weight measurements were taken on the weaning day (week 3), the day prior to treatment (week 8), and the day after treatment (week 11) by balances (MS3002ts/00, Mettler Toledo).

### 2.5. Behavioral Tests

Behavioral tests were started on the day after all treatments were over. All behavioral tests were performed during an active period of animals' light cycle (07:00–19:00). The behavior of rats was recorded by video analysis system (Shanghai Jiliang Software Technology Co., Ltd., Shanghai, China). The investigators for behavior test were blinded for groups. To avoid effects of learning and memory, rats were tested for the same paradigms only one time [34].

#### 2.5.1. Morris Water Maze (MWM)

The purpose of MWM test was to measure hippocampus-dependent spatial memory, as described before [35]. The diameter of swimming pool was 1.6 m and the height was 0.6 m. The pool was filled with water with a depth of 48 cm with 24 ± 1°C. The water was made opaque with the addition of a small quantity of nontoxic, water-based black paint. An escape platform (10 cm in diameter) was placed in the pool and the top of the platform was 1 cm submerged under the surface of the water. The swimming behavior of rats was recorded by a video camera located above the center of the swimming pool and was analyzed. Four visual cues used as spatial references for rats were located on the center of the 4 quadrant walls of the pool, including square, round, triangular, and star. The starting position, at which subjects were placed in the pool facing the wall, differed randomly across trials. The rats were trained for 5 consecutive days (4 trials/day) and they were allowed to swim until they found the platform or until 60 sec elapsed. If a rat found the platform within 60 sec, it was allowed to stay on the platform for 10 sec. If a rat failed to find the platform, it was guided to the platform and permitted to remain there for 10 sec. The trail interval among each quadrant of a rat was 15 minutes. At the end of the trial, rat was dried and replaced to the home cage. After 5 days of training, the escape platform was removed from the swimming pool. The test was on the sixth day. Every rat started swimming at the side of the pool opposite to the platform with the head facing the pool wall and was allowed to swim for 60 sec freely. The latency during the training days and the percent of distance spent in the target quadrant on test day were recorded.

#### 2.5.2. Fear Conditioning Test

Test procedure was used to measure fear conditioning to tone and context as described in [36]. On the first day, rats were initially placed into Context A made of an operant chamber, metal walls, and bars on floor to habituate for 2 minutes without being disturbed. During the 2 minutes, the baseline of freezing behavior was recorded. And then they were presented with a 10 sec 80-dB white noise which served as the conditioned stimulus (CS), and a 2 sec shock (1.0 mA) was followed. Five tone-shock pairings were repeated every 70 sec. 60 sec following the final shock, the rats were taken out of the chamber and returned to their home cage. On the second day, each rat was placed into Context B, a novel context made of black plastic walls, bedding on the floor, unscented. After 2 min of habituation, they were presented with an 8 min tone (80-dB), with no shock, and scored for freezing behavior, as a measure of fear. 48 hours later, the rats were returned to Context A and freezing behavior was recorded for a 5 min trial (no shock, no tone) to assess its response to the original conditioning context. The chamber of fear conditioning was cleaned with 70% ethanol before each rat test. Fear conditioning to tone is percentage of freezing during the first 4 min exposed to the tone/percentage of freezing during the Context B habituation period. Fear conditioning to context is percentage of freezing for the total time exposed to Context A on day 3/the baseline of freezing percentage.

### 2.6. In Vivo Electrophysiological Recordings for Long-Term Potentiation

Adult male rats were anesthetized with isoflurane (RWD, Shenzhen, China). Induced anesthesia concentration was 4%, and concentration was maintained at 2-3% and positioned on a stereotaxic frame (RWD, Shenzhen, China). The rats were placed on a heating pad to maintain body temperature. Two holes were drilled in the skull by using a dental drill for recording and stimulating electrodes. According to the rat brain atlas (Paxinos, Watson, 1986), the bipolar stimulating electrode was implanted into the CA3 area (3.8 mm posterior to bregma, 2.1 mm lateral to midline, and 1.5 mm ventral below dura) of the hippocampus, and the recording electrode was placed in the CA1 area (3.4 mm posterior to bregma, 2.5 mm lateral to midline, and 3.0 mm ventral below dura). Field excitatory postsynaptic potentials (fEPSPs) were recorded in hippocampus of the CA1 area through current stimuli to the stimulating electrode in CA3 area. Baseline stimuli were delivered at 0.1 Hz, and the evoked response was digitized (10K Hz) and analyzed by using the Cambridge Electronic Design 1401 (Cambridge, UK) and the software Spike2 (Cambridge, UK).The stimuli ranged from 0.05 to 1 mA to find the optimal stimulus intensity that evokes a response of half of its maximum amplitude. The baseline fEPSPs were recorded under single-pulse (monopolar pulses, 0.2 ms duration) stimulation for 30 minutes, and the LTP was induced by high frequency stimulation (HFS; two trains consisting of 100 pulses at 100 Hz; pulses interval, 10 ms; train interval, 30 s). The amplitudes of fEPSPs were recorded every 30 s for 60 mins after HFS. LTP was measured as the percentage of change of average fEPSPs slope at the last 10 minutes after HFS induction, comparing with the average fEPSPs slope of baseline.

### 2.7. Statistics

Data were presented as mean ± standard error of mean (SEM) and were analyzed by using SPSS 20.0 (Chicago, IL, USA) and GraphPad Prism version 5.0 (San Diego, CA, USA). One-way ANOVA was used to analyze multiple comparisons, and two-way ANOVA was used to analyze the results from MWM and fearing conditioning. Values of *P* < 0.05 were considered statistically significant.

## 3. Result

### 3.1. Both MS and Repeated EA Stimulation Did Not Alter Body Weight

To determine whether neonatal MS or repeated EA stimulation in young adult affects the nutritional development, animals in all subgroups were weighted on postnatal 3rd week (the weaning day), 8th week (the day before EA treatment), and the 11th week (the day after EA treatment). As shown in Figure 1(b), body weight at different time did not differ in control, MS, MS + EA, and MS + Sham-EA group (*P* > 0.05). These results demonstrated that both neonatal MS and repeat EA stimulation in this study had no effect on overall normal nutritional development of rats.

### 3.2. EA Improves Spatial Learning and Memory in Young Adult Rats with Neonatal MS

**Figure 2:**
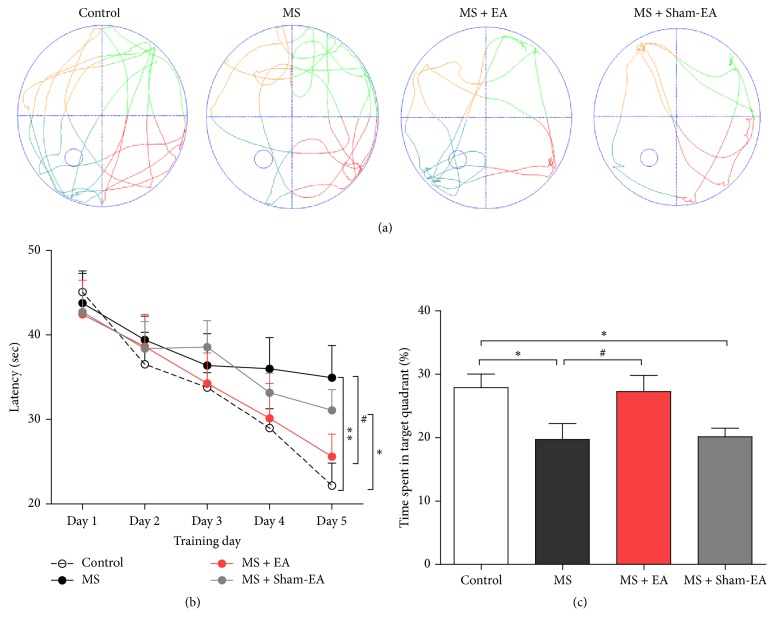
EA ameliorated spatial learning and memory deficits in rats with neonatal MS. (a) Representative swim paths in target quadrant from each group during the probe trials. The third quadrant is the target quadrant. (b) Increased latency in MS rats to reach the hidden platform in MWM, compared to control, and latency was reduced by repeating EA stimulation, but not MS + Sham-EA. *n* = 11/group; *F*_(3,40)_ = 3.731; *P* < 0.05, ^*∗*^*P* < 0.05, and ^*∗∗*^*P* < 0.01 versus control group; ^#^*P* < 0.05 versus MS group; two-way ANOVA. (c) Reduced percentage of time spent in target quadrant of rats in MS group, compared to rats in control, and percentage of time spent in platform quadrant was increased after EA treatment. *n* = 11/group. *F*_(3,40)_ = 4.047; *P* < 0.05, ^*∗*^*P* < 0.05 versus control group; ^#^*P* < 0.05 versus MS group; one-way ANOVA.

To assess effect of EA treatment on cognitive deficit in young adult rats that suffered maternal separation, rats were tested for spatial learning and memory with MWM, a widely used test in rodents known to require hippocampal function [37]. As shown in Figure 2(b), control group showed normal learning ability by showing gradually shorter escape latency to find the hidden platform during the MWM test; however, the latency was increased in MS group and MS + Sham-EA group compared to control group (*P* < 0.01, *P* < 0.05, resp.), indicating that ability of learning was compromised. In contrast, the latency in MS + EA group decreased significantly when compared to MS group (*P* < 0.01, Figure 2(b)). In probe trial after five days of training, rats were assessed regarding reference memory with target quadrant (Figures 2(a) and 2(c)) on the sixth day. Times spent in target quadrant of rats in MS group and MS + Sham-EA group were both less than control group (*P* < 0.05, *P* < 0.05, resp.), but time spent in target quadrant of rats in MS + EA group was increased compared with MS group (*P* < 0.05) and had no difference with control group (*P* > 0.05). These results indicated that repeated EA stimulation improved spatial learning and reference memory in young adult rats with neonatal MS.

### 3.3. EA Attenuated Contextual Fear Memory Impairment in Rats with Neonatal MS

**Figure 3:**
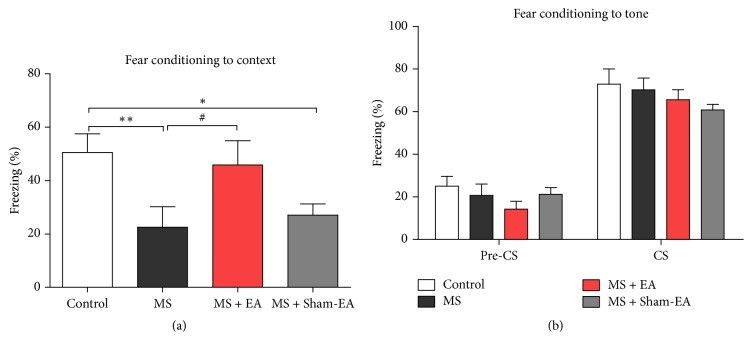
Effect of EA on performance in the fear conditioning task. (a) EA ameliorated impairment in contextual fear memory in rats with neonatal MS, *n* = 11/group; *F*_(3,40)_ = 3.660; *P* < 0.05, ^*∗*^*P* < 0.05, and ^*∗∗*^*P* < 0.01 versus control group; ^#^*P* < 0.05 versus MS group; one-way ANOVA. (b) There was no impairment in fear conditioning to tone in rats suffered maternal separation. CS represents condition stimulus, such as sound combined with an aversive unconditioned stimulus (foot shock); pre-CS represents a 120 sec pause without stimulation. *n* = 11/group; *F*_(3,40)_ = 0.9171; *P* > 0.05; two-way ANOVA.

To further demonstrate that EA could attenuate hippocampal-dependent cognitive impairment in rats with neonatal MS, we then explored performance of rat in the contextual fear conditioning task. As shown in Figure 3(a), we found that rats in MS group and MS + Sham-EA group exhibited decreased levels of freezing compared to controls (*P* < 0.01, *P* < 0.05, resp.). However, the level of freezing in MS + EA group was similar to control group (*P* > 0.05), demonstrating normal contextual fear memory. We next assessed cued fear conditioning task, which was generally believed to be largely dependent on amygdala, but not hippocampus [38]. As shown in Figure 3(b), no differences were found in percentage of freezing to the tone among all groups (*P* > 0.05). Together, the results from fear conditioning and MWM tests suggested that neonatal MS may specifically impair the hippocampal-dependent cognitive function, while EA ameliorated the deficit in young adult period.

### 3.4. EA Improved the Impaired LTP at SC-CA1 Synapse of Rats with Neonatal MS

**Figure 4:**
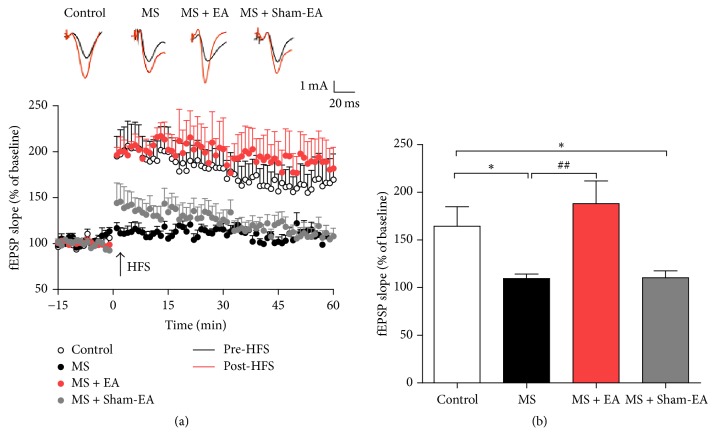
EA enhanced LTP in the hippocampus of young adult rats with neonatal MS. (a) Averaged time course changes in fEPSPs slope induced by HFS in hippocampal of rats. (b) Percentage of change in magnitude of LTP (fEPSPs slope average at last 10 minutes after HFS). *n* = 5-6 rats/group; *F*_(3,19)_ = 6.282; *P* < 0.01, ^*∗*^*P* < 0.05 versus control group; ^##^*P* < 0.01 versus MS group; one-way ANOVA.

To better directly assess the role of EA treatment in altering hippocampal synaptic plasticity of cognitive deficit rat, electrophysiological study used to record in vivo LTP induced by HFS at SC-CA1 synapses in the hippocampus was performed. As shown in Figure 4, fEPSPs were measured in the CA1 area of the dorsal hippocampus and the LTP magnitude was quantified in groups. We found that the LTP amplitude was largely suppressed in MS and MS + Sham-EA group compared with control group (*P* < 0.05, *P* < 0.05, resp.). Intriguingly, there was no difference between MS + EA group and control group.

## 4. Discussion

In this study, we modeled rat maternal separation which was observed commonly in society. The major findings of our study are as follows. First, we found that rat with neonatal MS exhibited normal weight gain and significant cognitive deficit in MWM and contextual fear conditioning test in adulthood (Figures 2 and 3). Intriguingly, after 3-week EA intervention in young adulthood, behavior deficits of young adult rats with maternal separation in early-life stage were ameliorated significantly (Figures 2 and 3). This indicated the efficacy of EA intervention on cognitive deficits in rats exposed to the neonatal MS. Moreover, this paper provided evidence that in vivo LTP induced by the HFS at hippocampal SC-CA1 synapses was suppressed in rat with neonatal MS, and EA intervention could restore LTP induction significantly (Figure 4), which identified straightforward function of EA intervention in regulating hippocampal synaptic plasticity in vivo.

The present study showed normal weight gain of young adult rats with MS. This result agreed with previous studies examining maternal separation on rats during PND2–9 or PND10–17 [39]. Some previous studies showed that repeated maternal separation altered body weight in offspring [40, 41], which may be related to variation of MS protocol (longer versus shorter time separation), species of animals, or the different postnatal periods of MS [42]. Moreover, EA stimulation in young adulthood does not affect the weight gain of rats with MS, compared to the MS or control group (Figure 1(b)). Thus, all the interpretation to the following results in this study may not be secondary to nutritional development by MS or EA intervention.

Hippocampus plays an important role in spatial information processing in both rodents and humans [29]. Morris water maze and other tasks were used to assess spatial memory related to hippocampus [43, 44]. In the present study, we demonstrated that rats with neonatal MS exhibited significant spatial learning and memory deficits which agree with previous studies [39, 45, 46]. It is generally believed that contextual fear conditioning is dependent on hippocampus, while cued fear conditioning largely involves amygdala [47]. We found cognitive deficit significantly in contextual fear conditioning test, but not cured fear conditioning test (Figure 3). The result supported the point that MS in early age impaired hippocampal-dependent cognitive function in adulthood.

Our result provided evidence for the first time that EA intervention at GV 20 and GV 29 significantly enhanced the impaired learning and memory ability in rat model exposed to the neonatal MS, which was similar to studies by using EA intervention in other cognitive deficit researches. A study found that EA intervention ameliorated ethanol-induced impairments of spatial learning and memory [48]. Huang et al. proved that ischemic stroke which accompanied memory in rat was improved by EA treatment at GV 20 and* Shenting* (DU24) acupoints [49]. Ye et al. reported that acupuncture at* Zusanli* (ST36) and GV 20 remarkably reversed cognitive deficits in 2-vessel occlusion model rats [50]. EA improved memory was also observed in neurological diseases such as MCI, Parkinson's disease, and Alzheimer disease [51]. These studies in animal models support the clinical data that acupuncture is promising for the treatment of cognitive deficits in various diseases [19–23].

The present results also showed that significant impaired LTP in rats with neonatal MS and EA treatment, but not Sham-EA, significantly enhanced the increasing of the slope of fEPSPs. LTP at hippocampal SC-CA1 synapse is widely considered the cellular mechanism that underlies learning and memory [28]. Previous studies suggest that chronic stress in early life was proven to impair LTP, resulting in spatial and fear related learning and memory [45, 52]. And it has been shown that EA could enhance LTP at SC-CA1 synapse in the hippocampus to improve cognitive deficits [27, 53]. These suggest that EA may improve spatial or fear learning and memory via enhancing LTP ability. However, the mechanisms of EA on learning and memory ability are not understood. In recent years, converging evidence has shown that one of the most important underlying mechanisms for improving learning memory is via modulating hippocampus synaptic plasticity [29, 54]. In ischemic stroke animals model, manual acupuncture at ST 36 and EA at GV 20 improves cognitive hippocampus function by modulating cAMP/PKA/CREB [55] signaling pathway and by reducing the expression of NR1-TRPV1 [56], thus reducing deficits related to LTP. In vascular dementia rat model, cognition and hippocampal synaptic plasticity were improved by acupuncture via activating D1/D5 receptors [21]. These results agree with our observations in this study, which suggests that EA ameliorated learning and memory deficit in maternal separation animals via restoration of hippocampal LTP induction at SC-CA1 synapses. However, confirmation of the underlying molecular mechanisms of EA enhanced hippocampal neural plasticity and behavior deficit in adult induced by neonatal MS still await the results of further studies.

## 5. Conclusions

In conclusion, EA ameliorated learning and memory deficit in young adult rats that experienced neonatal maternal separation stress via restoration of hippocampal LTP induction at SC-CA1 synapses. EA at GV 20 and GV 29 may be of therapeutic value for its importance for the memory and cognitive dysfunction resulting from early stage stress.
